# Supplementary material for: Mapping the evolution of fertility support policies in China: A content and instrumental analysis
Source: PLoS One. 2025 Oct 9;20(10):e0332137. doi: 10.1371/journal.pone.0332137 (PMC12510515; doi:10.1371/journal.pone.0332137)
Supplement: S1 Appendix — (ZIP) [file pone.0332137.s001.zip › S1 Appendix. 226 original policy documents/194-国家发展改革委、教育部关于印发《建设产教融合型企业实施办法(试行)》的通知(FBM-CLI-4-331051).doc]

国家发展改革委、教育部关于印发《建设产教融合型企业实施办法(试行)》的通知

**发布部门：** [国家发展和改革委员会(含原国家发展计划委员会、原国家计划委员会)](https://www.pkulaw.com/law/chl?Aggs.IssueDepartment=60301) [教育部](https://www.pkulaw.com/law/chl?Aggs.IssueDepartment=60203) [机构沿革](javascript:void(0))

**发文字号：**发改社会〔2019〕590号

**发布日期：**2019.03.28

**实施日期：**2019.03.28

**时效性：** [现行有效](https://www.pkulaw.com/law/chl?Aggs.TimelinessDic=01)

**效力级别：** [部门规范性文件](https://www.pkulaw.com/law/chl?Aggs.EffectivenessDic=XE0302)

**法规类别：** [教育综合规定](https://www.pkulaw.com/law/chl?Aggs.Category=09101)

国家发展改革委、教育部关于印发《建设产教融合型企业实施办法（试行）》的通知
（发改社会〔2019〕590号）

各省、自治区、直辖市及计划单列市、新疆生产建设兵团发展改革委、教育厅（教委、教育局），各中央企业：
　　经国务院职业教育工作部际联席会议审议通过，现将《建设产教融合型企业实施办法（试行）》印发给你们，请认真遵照执行。

国家发展改革委
教　　育　　部
2019年3月28日

　　建设产教融合型企业实施办法（试行）

　第一章　总则

　　**第一条**　为深入贯彻党的十九大和全国教育大会精神，完善职业教育和培训体系，深化产教融合、校企合作，充分发挥企业在技术技能人才培养和人力资源开发中的重要主体作用，根据《加快推进教育现代化实施方案（2018-2022年）》《国家职业教育改革实施方案》要求，制定本办法。

　　**第二条**　本办法规定的产教融合型企业是指深度参与产教融合、校企合作，在职业院校、高等学校办学和深化改革中发挥重要主体作用，行为规范、成效显著，创造较大社会价值，对提升技术技能人才培养质量，增强吸引力和竞争力，具有较强带动引领示范效应的企业。

　　**第三条**　建设产教融合型企业，按照政府引导、企业自愿、平等择优、先建后认、动态实施的基本原则开展。

　　**第四条**　国家发展改革委、教育部会同相关部门共同负责建设产教融合型企业工作的政策统筹、组织管理和监督实施。
　　国家发展改革委、教育部将建设产教融合型企业纳入深化产教融合改革的整体制度安排，在国家产教融合建设试点中统筹推进，提出产教融合型企业的建设培育条件、认证标准、评价办法，指导各地建立产教融合型企业建设信息服务平台和信息储备库，做好建设产教融合型企业的日常管理工作。
　　人力资源社会保障部、工业和信息化部、财政部、国务院国资委等相关部门根据职能职责，配合做好产教融合型企业建设的政策支持和推进实施工作。
　　省级（含计划单列市，下同）发展改革部门、教育行政部门共同负责区域内建设产教融合型企业的组织申报、复核确认、建设培育、认证评价和日常管理工作。

　第二章　建设培育条件

　　**第五条**　在中国境内注册成立的企业，通过独资、合资、合作等方式，利用资本、技术、知识、设施、管理等要素，依法举办或参与举办职业教育、高等教育，在实训基地、学科专业、教学课程建设和技术研发等方面稳定开展校企合作，并具备以下条件之一。
　　1. 独立举办或作为重要举办者参与举办职业院校或高等学校；或者通过企业大学等形式，面向社会开展技术技能培训服务；或者参与组建行业性或区域性产教融合（职业教育）集团。
　　2.承担现代学徒制和企业新型学徒制试点任务；或者近3年内接收职业院校或高等学校学生（含军队院校专业技术学员）开展每年3个月以上实习实训累计达60人以上。
　　3.承担实施1＋X证书（学历证书＋职业技能等级证书）制度试点任务。
　　4. 与有关职业院校或高等学校开展有实质内容、具体项目的校企合作，通过订单班等形式共建3个以上学科专业点。
　　5.以校企合作等方式共建产教融合实训基地，或者捐赠职业院校教学设施设备等，近3年内累计投入100万元以上。
　　6. 近3 年内取得与合作职业院校共享的知识产权证明（发明专利、实用新型专利、软件著作权等）。

　　**第六条**　重点建设培育主动推进制造业转型升级的优质企业，以及现代农业、智能制造、高端装备、新一代信息技术、生物医药、节能环保、新能源、新材料以及研发设计、数字创意、现代交通运输、高效物流、融资租赁、工程咨询、检验检测认证、电子商务、服务外包等急需产业领域企业，以及养老、家政、托幼、健康等社会领域龙头企业。优先考虑紧密服务国家重大战略，技术技能人才需求旺盛，主动加大人力资本投资，发展潜力大，履行社会责任贡献突出的企业。主营业务为教育培训服务的企业原则上不纳入建设培育范围。

　　**第七条**　企业无重大环保、安全、质量事故，具有良好信用记录，无涉税等违法违规经营行为。

　第三章　建设实施程序

　　**第八条**　产教融合型企业的建设实施由国家发展改革委、教育部会同相关部门结合开展国家产教融合建设试点统筹部署。

　　**第九条**　省级行政区域内的企业按照自愿申报、复核确认、建设培育、认证评价等程序开展产教融合型企业建设实施。
　　1. 自愿申报。省级发展改革、教育行政部门会同有关部门和有关城市人民政府，结合开展国家产教融合建设试点有关要求，组织辖区内符合建设培育条件的企业按照自愿申报并提交证明材料。省级发展改革、教育行政部门应建立产教融合型企业建设信息服务平台，实行网上申报、网上受理、网上办理。
　　2. 复核确认。省级发展改革、教育行政部门组织行业主管部门和行业组织等有关方面，对辖区内申报企业进行复核，符合条件的纳入建设培育范围，列入产教融合型企业建设信息储备库，向全社会公示。
　　3.建设培育。国家发展改革委、教育部结合组织开展国家产教融合建设试点，指导各地开展产教融合型企业建设培育，鼓励支持企业多种方式参与举办教育，深度参与“引企入教”改革，推动学生到企业实习实训制度化、规范化，发挥企业办学重要主体作用，建立以企业为主体的协同创新和成果转化机制，提高企业职工在岗教育培训覆盖水平和质量。各地要有针对性地制定具体可操作的培育举措。建设培育企业要制订并向全社会公开发布产教融合、校企合作三年规划，并需经过至少1年的建设培育期。
　　4. 认证评价。在各地推进试点工作基础上，教育部、国家发展改革委研究制定产教融合型企业认证标准和评价办法，指导省级政府出台具体实施办法，建立产教融合型企业认证目录，对纳入产教融合型企业建设信息储备库的企业进行逐年、分批认证，并定期向全社会公布推介。支持开展产教融合型企业第三方评价。

　　**第十条**　中央企业、全国性特大型民营企业整体申报建设国家产教融合型企业，由国家发展改革委、教育部会同相关部门部署实施。上述企业的下属企业或分支机构建设产教融合型企业的，按照属地管理原则实施。

　第四章　支持管理措施

　　**第十一条**　纳入产教融合型企业建设信息储备库的建设培育企业，省级政府要落实国家支持企业参与举办职业教育的各项优惠政策，实行定期跟踪、跟进服务、确保落地；结合开展产教融合建设试点，在项目审批、购买服务、金融支持、用地政策等方面对建设培育企业给予便利的支持。

　　**第十二条**　进入产教融合型企业认证目录的企业，给予“金融＋财政＋土地＋信用”的组合式激励，并按规定落实相关税收政策。激励政策与企业投资兴办职业教育、接收学生实习实训、接纳教师岗位实践、开展校企深度合作、建设产教融合实训基地等工作相挂钩，具体办法另行制定。

　　**第十三条**　进入产教融合型企业认证目录的企业，建立实施推进产教融合工作年报制度，报省级发展改革、教育行政部门备案，并按程序向全社会公示。

　　**第十四条**　进入产教融合型企业认证目录的企业，每3年由省级发展改革、教育行政部门对其进行资格复核，复核合格的继续确认其产教融合型企业资格，不合格的不再保留产教融合型企业资格。

　　**第十五条**　进入产教融合型企业认证目录的企业，有下列情况之一的，即取消其资格，且5年内不得再行申报。
　　1. 在申请认证、年度报告或考核过程中弄虚作假，故意提供虚假不实信息的。
　　2. 在资格期内发生重大环保、安全、质量事故，存在违法违规经营行为的。
　　3. 侵犯学生人身权利或其他合法权利的。
　　4. 列入失信联合惩戒对象名单的。

　第五章　附则

　　**第十六条**　本办法由国家发展改革委、教育部负责解释。

　　**第十七条**　本办法自发布之日起施行。

© 北大法宝：（[www.pkulaw.com](https://www.pkulaw.com/)) 专业提供法律信息、法学知识和法律软件领域各类解决方案。北大法宝为您提供丰富的参考资料，正式引用法规条文时请与标准文本核对。
欢迎查看所有[产品和服务](https://www.pkulaw.com/helps/2.html)。法宝快讯：[如何快速找到您需要的检索结果？](https://www.pkulaw.com/helps/70.html)[法宝](https://www.pkulaw.com/helps/69.html)V6有何新特色？

扫描二维码阅读原文

**原文链接：**<https://www.pkulaw.com/chl/cb28b9104081f2dabdfb.html>
